# Enhancing Electronic Nose Performance by Feature Selection Using an Improved Grey Wolf Optimization Based Algorithm

**DOI:** 10.3390/s20154065

**Published:** 2020-07-22

**Authors:** Chao Zhang, Wen Wang, Yong Pan

**Affiliations:** 1 Institute of Acoustics, Chinese Academy of Sciences, Beijing 100190, China; zhangchao1@mail.ioa.ac.cn; 2 School of Electronic, Electrical and Communication Engineering, University of Chinese Academy of Sciences, Beijing 100190, China; 3 State Key Laboratory of NBC Protection for Civilian, Beijing 102205, China; panyong71@sina.com.cn

**Keywords:** feature selection, grey wolf optimization, electronic nose, classification, wrapper method

## Abstract

Electronic nose is a kind of widely-used artificial olfactory system for the detection and classification of volatile organic compounds. The high dimensionality of data collected by electronic noses can hinder the process of pattern recognition. Thus, the feature selection is an essential stage in building a robust and accurate model for gas recognition. This paper proposed an improved grey wolf optimizer (GWO) based algorithm for feature selection and applied it on electronic nose data for the first time. Two mechanisms are employed for the proposed algorithm. The first mechanism contains two novel binary transform approaches, which are used for searching feature subset from electronic nose data that maximizing the classification accuracy while minimizing the number of features. The second mechanism is based on the adaptive restart approach, which attempts to further enhance the search capability and stability of the algorithm. The proposed algorithm is compared with five efficient feature selection algorithms on three electronic nose data sets. Three classifiers and multiple assessment indicators are used to evaluate the performance of algorithm. The experimental results show that the proposed algorithm can effectively select the feature subsets that are conducive to gas recognition, which can improve the performance of the electronic nose.

## 1. Introduction

Feature selection is an important technique in the applications of pattern recognition. In practical application, there are usually too many redundant features in the the data, which will greatly affect the classification accuracy and computational complexity. In addition, in order to eliminate the influence of redundant features on classification process, the feature selection plays an important role in reducing the dimension of data, improving the accuracy of the model and helping us have insight into the data more deeply [1].

Feature selection can be roughly divided into three categories: filter, wrapper, and embedded [2]. The filter method sorts the features according to predefined criteria, and the feature selection process is independent of the classification. The wrapper method wraps the classifier in the search algorithm and it is guided by the objective function. In the embedded method, the selection of variables is integrated into the training process.

Electronic nose is a kind of system for the detection and classification of volatile organic compounds by imitating human olfactory [3,4]. The electronic noses are composed of a gas sensor array, and the collected data are classified by the pattern recognition algorithm [5]. In recent years, electronic noses have played an important role in various fields, such as environmental monitoring, food inspection, biomedical diagnosis, etc. [6,7,8,9,10,11]. Too many redundant features are produced by the large number of gas sensors and lots of feature extraction functions in the electronic nose system, which seriously affects the accuracy of classification. Thus, many researchers have put forward the algorithm for feature selection of gas sensor data and achieved positive results [12,13,14,15,16,17].

Optimization is a process of finding the solution that optimizes the specific objective under certain conditions. Traditional optimizations still have some limitations in solving the problems, and meta-heuristic algorithms make up for them. Meta-heuristic algorithms are usually inspired by the special behavior or evolution process of biological groups in nature (birds, bat, bee, wolves, fish, animals, etc.) [18], and a multitude of algorithms have been proposed by imitating the above behaviors [19,20,21,22]. The binary version of meta-heuristic algorithms have a positive performance in solving binary optimization problems. Thus, there are many different binary meta-heuristic algorithms have been proposed to solve the problem of feature selection and achieved excellent results [23,24,25,26].

Grey wolf optimizer (GWO) is a newly introduced meta-heuristic algorithm with positive search capability. Many different improved binary GWO algorithms have been proposed to solve the problem of feature selection in different applications [27,28,29,30,31]. However, they have not been applied to gas recognition and they suffer from the problem of local optimization which reduce the search capability. In order to rectify the above problems, the adaptive restart GWO (ARGWO) with two novel binary transform approaches is proposed, and the main contributions of this paper include the following:

- This paper applies the grey wolf optimization based algorithm to the feature selection of electronic nose data for the first time. Two novel transform approaches and adaptive restart approach are employed for the proposed algorithm to enhance classification accuracy and reduce the dimension of electronic nose data, which further enhance the performance of electronic nose.
- The proposed algorithm is compared with other classical feature selection algorithms over three electronic nose datasets in multiple assessment indicators, and the experimental results show that the proposed algorithm is superior to other algorithms over all datasets, which proves that the proposed algorithm can be applied to different types of electronic nose and achieve positive results.
- The effect of two proposed transform approaches and adaptive restart approach are investigated in multiple assessment indicators, which proves that the two mechanisms are helpful in selecting better features of odor and enhancing the accuracy of final gas recognition.

The organization of this paper is as follows: Section 2 provides a description of the proposed algorithm. Section 3 introduces the data set and the feature extraction methods that are used in the experiment. The experimental results are discussed in Section 4. Finally, Section 5 concludes the paper.

## 2. Methodology

In this section, the proposed algorithm is expected to solve the feature selection problem of gas sensor data. The main parts of the algorithm are original grey wolf optimization, evaluation, binary transform approach, and adaptive restart approach, which will be described in more detail in the following subsections.

### 2.1. Grey Wolf Optimization

Grey wolves are social animals with special skills in catching prey. Wolves can catch prey in the shortest time through the cooperation and strict grading. In the pack of wolves, the leader is called alpha, and alpha wolves are responsible for making decisions on predation. Beta wolves are on the second level and they are responsible for assisting alpha wolves to make decisions. The delta wolves have to submit alpha wolves and beta wolves, but they can command the omega wolves. They are responsible for monitoring the surrounding environment and warning groups in case of danger. Omega wolves have to follow the command of other levels.

In the mathematical model of GWO, α, β, and δ represent the first, second, and third optimal solution. The rest search agents are collectively referred to as ω. The model is guided by α, β, and δ. The grey wolves will gradually approach the prey and surround it while hunting [32]. This behavior can be modeled mathematically as follows:

(1) $$\vec{D}=\left|\vec{C}\cdot {\vec{X}}_{p}\left(t\right)-\vec{X}\left(t\right)\right|$$

(2) $$\vec{X}\left(t+1\right)={\vec{X}}_{p}\left(t\right)+\vec{A}\cdot \vec{D}$$

(3) $$\vec{A}=2a\cdot \vec{rand1}-a$$

(4) $$\vec{C}=2\cdot \vec{rand2}$$

where ${\vec{X}}_{p}\left(t\right)$ and $\vec{X}\left(t\right)$ represent the position vector of prey and grey wolf in the iteration *t*, $\vec{rand1}$ and $\vec{rand2}$ are random vectors in the range of 0 to 1, *a* is the number that decreases linearly from 2 to 0 during the whole iteration according to Equation (5):

(5) $$a=2-t\times \frac{2}{M_{Iter}}$$

where $M_{Iter}$ is the maximum iterations of the algorithm. It is assumed that α, β, and δ are the first three best solutions in the process of the searching optimal solution. Thus, the three wolves with the first three minimum fitness value are retained as α, β, and δ during each iteration, which will guide the model to update the position of other search agents. This process can be represented by the mathematical model as follows:

(6) $$\vec{X}\left(t+1\right)=\frac{\vec{X_{1}}+\vec{X_{2}}+\vec{X_{3}}}{3}$$

(7) $$\vec{X_{1}}=|\vec{X_{\alpha }}-\vec{A_{1}}\cdot \vec{D_{\alpha }}|\vec{D_{\alpha }}=\left|\vec{C_{1}}\cdot \vec{X_{\alpha }}-\vec{X}\right|$$

(8) $$\vec{X_{2}}=|\vec{X_{\beta }}-\vec{A_{2}}\cdot \vec{D_{\beta }}|\vec{D_{\beta }}=\left|\vec{C_{2}}\cdot \vec{X_{\beta }}-\vec{X}\right|$$

(9) $$\vec{X_{3}}=|\vec{X_{\delta }}-\vec{A_{3}}\cdot \vec{D_{\delta }}|\vec{D_{\delta }}=\left|\vec{C_{3}}\cdot \vec{X_{\delta }}-\vec{X}\right|$$

α, β, and δ will keep searching for prey during the process of hunting, and Algorithm 1 outlines the grey wolf optimization (GWO) algorithm.
**Algorithm 1** Grey wolf optimization1:Initialize the number of iterations for optimization $N_{iter}$2:Initialize the positions of n grey wolves $X_{i}$, i= 1, 2, …, *N*3:Calculate the fitness value of each grey wolf4:Choose the best three grey wolves as $X_{\alpha }$, $X_{\beta }$, $X_{\delta }$ base on there fitness5:t←0.6:**while***t* < $N_{iter}$ **do**7:    Update the position of the wolves using to Equation (6)8:    Update α, *A*, and *C*9:    Calculate the fitness of each grey wolf10:   Update the first three grey wolves $X_{\alpha }$, $X_{\beta }$, $X_{\delta }$11:   t←t+1.12:**end while**13:**return**$best\;fitness,X_{\alpha }$

### 2.2. Evaluation

Fitness value is the evaluation criteria for searching the optimal solution. The fitness function needs to ensure that the calculated solution can have high classification accuracy in different classifiers, which is used for guiding the algorithm to find the optimal solution.

K-nearest neighbor (KNN) is a commonly used classification method. The test samples are classified with KNN by analyzing the categories of K training samples closest to the test samples in feature space. KNN is easy to implement and has great performance in multi-class classification, hence it was used to calculate the fitness of the proposed algorithm.

In order to find the optimal feature subset, the evaluation of selected feature subsets must be considered from the following two aspects:

- Maximum classification accuracy
- Minimum number of features

Considering the above two factors, the fitness function is as shown in Equation (10):

(10) $$f=\alpha P_{R}\left(D\right)+\beta \frac{\left|S\right|}{\left|F\right|}$$

where *f* is the fitness value. $P_{R}\left(D\right)$ is the error rate of test set with selected features under decision *D*. |F| and |S| are the length of the original eigenvector and the eigenvector with selected features. α and β are the weights for balancing the classification accuracy and eigenvector length, where alpha∈ [0,1] and beta=1−α. The experimental data set was divided into train set and test set. 10-fold cross-validation was used to train classification models on the train set to prevent over fitting, and the model with the best performance was used to calculate the error rate on the test set.

### 2.3. Binary Transform Approach

Each dimension of the solution obtained by the original GWO is a continuous value. Because of the particularity of feature selection problem, the solution needs to be limited to the binary (0,1) value. The S-shaped and V-shaped transform functions are usually used to convert from decimal to binary [33]. Two new approaches for mapping search agents to binary vectors will be introduced in the following.

#### 2.3.1. Approach1

In this approach, the main function can be formulated as shown in Equation (11):

(11) $$x_{binary}^{d}=\left\{\begin{array}{ccc} 1 & \; & \frac{x_{1}^{d}+x_{2}^{d}+x_{3}^{d}}{3}\geq 0.5\\ 0 & \; & \frac{x_{1}^{d}+x_{2}^{d}+x_{3}^{d}}{3}<0.5 \end{array}\right.$$

where $x_{binary}^{d}$ is the binary value of each search agent in dimension d, $x_{1}^{d}$, $x_{2}^{d}$, $x_{3}^{d}$ are calculated using Equations (12)–(14).

(12) $$x_{1}^{d}=\left\{\begin{array}{ccc} 1 & \; & GDT1^{d}\geq rand\\ 0 & \; & GDT1^{d}<rand \end{array}\right.GDT1^{d}=\int_{-\infty }^{x_{\alpha }^{d}-A_{1}^{d}D_{\alpha }^{d}}\;\frac{1}{\sqrt{2\pi }}exp\left(-\frac{x^{2}}{2}\right)\;dx$$

where rand is a random number between 0 and 1, $x_{\alpha }^{d}$ is the value of alpha wolf in dimension *d*, $A_{1}^{d}$, and $D_{\alpha }^{d}$ are calculated using Equations (3) and (7).

(13) $$x_{2}^{d}=\left\{\begin{array}{ccc} 1 & \; & GDT2^{d}\geq rand\\ 0 & \; & GDT2^{d}<rand \end{array}\right.GDT2^{d}=\int_{-\infty }^{x_{\beta }^{d}-A_{2}^{d}D_{\beta }^{d}}\;\frac{1}{\sqrt{2\pi }}exp\left(-\frac{x^{2}}{2}\right)\;dx$$

where rand is a random number between 0 and 1, $x_{\beta }^{d}$ is the value of beta wolf in dimension *d*, $A_{2}^{d}$ and $D_{\beta }^{d}$ are calculated using Equations (3) and (8).

(14) $$x_{3}^{d}=\left\{\begin{array}{ccc} 1 & \; & GDT3^{d}\geq rand\\ 0 & \; & GDT3^{d}<rand \end{array}\right.GDT3^{d}=\int_{-\infty }^{x_{\delta }^{d}-A_{3}^{d}D_{\delta }^{d}}\;\frac{1}{\sqrt{2\pi }}exp\left(-\frac{x^{2}}{2}\right)\;dx$$

where rand is a random number between 0 and 1, $x_{\delta }^{d}$ is the value of delta wolf in dimension *d*, $A_{3}^{d}$ and $D_{\delta }^{d}$ are calculated using Equations (3) and (9).

#### 2.3.2. Approach2

In this approach, the effect of position vector on transformation is also considered, and the main function can be formulated as shown in Equation (15)

(15) $$x_{binary}^{d}=\left\{\begin{array}{ccc} 1 & \; & NBT^{d}\geq rand\\ 0 & \; & NBT^{d}<rand \end{array}\right.$$

where $x_{binary}^{d}$ is the binary value of each search agent in dimension *d*, rand is a random number between 0 and 1, $NBT^{d}$ is a continuous value between 0 and 1 which is calculated from the position vector and the value of dimension *d* as in Equation (16).

(16) $$NBT^{d}=\frac{x_{s}^{d}-x_{smin}}{x_{smax}-s_{smin}}$$

where $x_{s}^{d}$ is the value of position vector in dimension *d*, which is calculated by the original GWO.

### 2.4. Adaptive Restart GWO

Original GWO has a certain probability of falling into local optimum, which affects the search capability [34], and restart is an exceedingly economic strategy when the algorithm falls into complex problems [35]. Thus, the adaptive restart method is proposed to enhance the search capability of the algorithm. When the minimum fitness of the iteration t+1 is greater than or equal to the minimum fitness of the iteration *t*, we will harbor the idea that the search process has already or has a tendency to fall into the local optimum, and a slice of search agents will be reinitialized randomly. The adaptive restart approach emphasizes the dynamic reinitialization of search agent according to the optimal fitness of each iteration. The number of randomly reinitialized search agents is calculated by Equation (17).

(17) $$N_{AR}=round\left(N_{s}\times f_{t}\right)$$

where $f_{t}$ is the minimum fitness value of the iteration *t*. $N_{s}$ is the number of search agent. round represents the rounding operation. According to Equation (10), we can find that a higher value of fitness means a higher error rate or a larger number of selected features, which represents a poorer search effect in each iteration. Because fitness ∈ [0,1], the number of restarted search agents will vary from 0 to $N_{s}$ according to the search effect. Finally, the overall pseudocode of the ARGWO with two proposed binary transform approaches (ARGWO1 and ARGWO2) can be found in Algorithm 2.
**Algorithm 2** Adaptive restart GWO1:Initialize the number of iterations for optimization $N_{iter}$2:Initialize the positions of n grey wolves $X_{i}$, i=1,2,…,N3:Calculate the fitness value of each grey wolf4:Choose the best three grey wolves as $X_{\alpha }$, $X_{\beta }$, $X_{\delta }$ base on there fitness5:t←0.6:$fitness_{N}\leftarrow$ the best fitness calculated from initialized wolves7:**while**$t<N_{iter}+1$**do**8:    Update the position of the each wolf using Equation (6)9:    Update α, *A*, and *C*10:  Update $x_{1}$, $x_{2}$, and $x_{3}$ using Equations (12)–(14) or get the position vector of each wolf11:  Transform each wolf’s position into a binary vector using Equation (11) and $x_{1}$, $x_{2}$, $x_{3}$ or Equation (15) and wolf’s original position vector.12:    Calculate the fitness of each Wolf13:    $fitness_{L}\leftarrow fitness_{N}$14:    $fitness_{N}\leftarrow$ best fitness in iteration *t*15:    **if** $fitness_{N}\geq fitness_{L}$ **then**16:        calculate $N_{restart}$ using Equation (17).17:        select $N_{restart}$ wolves randomly from all search agents to reinitialize18:    **end if**19:    Update the first three grey wolves $X_{\alpha }$, $X_{\beta }$, $X_{\delta }$ base on fitness20:    t←t+1.21:**end while**22:**return**$best\;fitness,X_{\alpha }$

## 3. Datasets and Feature Extraction

Three gas sensor data sets in different application domains were used in this experiment, which will be described in more detail in the following subsections.

### 3.1. Dataset1

Dataset1 is the sensor array data collected by Vergara et al., which was publicly available in UCI Machine Learning Repository for detecting different gases [36,37]. Dataset1 contains 13,910 samples collected by 16 chemical sensors (TGS2600, TGS2602, TGS2610, and TGS2620 four of each), which were exposed to six different concentrations of gas (Ammonia, Acetaldehyde, Acetone, Ethylene, Ethanol, and Toluene). The information of the dataset1 is presented in Table 1.

Two distinct types of features were extracted from the response signal:

- The features defined as the maximal resistance change relative to the baseline and the DR normalized version.
- The features reflecting the increase/decrease transient part of the sensor response in the whole measurement process, which can be formulated as shown in Equation (18):

(18) y[k]=(1−α)y[k−1]+α(R[k]−R[k−1])

where R[k] is the resistance measured of each sensor at time *k*, α is a parameter between 0 and 1 to smooth the scalar. Six features of rising and falling stages of the sensor response were extracted by using three different alpha values (0.1, 0.01, 0.001). Thus, for each sensor, eight features were extracted, and the sample of 128-dimensional eigenvector was formed (16 sensors × 8 features).

### 3.2. Dataset2

**Figure 1:**
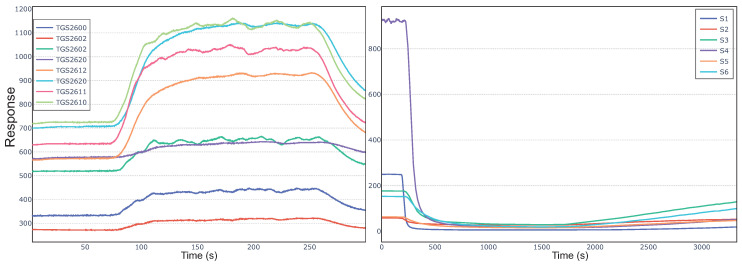
Typical sensor response curves in two datasets. **Left**: dataset2; **Right**: dataset3.

Dataset2 is the time series data collected by the chemical detection platform composed of eight chemo resistive gas sensors (TGS2611, TGS2612, TGS2610, TGS2600, two TGS2602, and two TGS2620) for detecting mixtures of Ethylene with Methane or Carbon Monoxide, and it was publicly available in UCI Machine Learning Repository [38]. There are 180 samples in dataset2, in order to reduce the difficulty of classification, and the pattern recognition of dataset2 was regarded as a binary classification problem. The example of the time series of dataset2 is outlined in Figure 1(left).

It is necessary to extract the hidden features contained in the gas sensor data in order to improve the classification accuracy [39]. For each sensor, 16 features were extracted, and the sample of 128-dimensional eigenvector was formed (8 sensors × 16 features). The feature extraction methods applied to dataset2 are outlined in Table 2.

### 3.3. Dataset3

Dataset3 is a time series dataset for the detection of wine quality [40,41]. Six gas sensors (Table 3) were used to detect different quality wines (high quality, medium quality, low quality) and ethanol, and 300 samples were collected. The example of the time series of dataset3 is outlined in Figure 1(right). The same feature extraction methods were used in dataset3, and the details can be seen in Table 2. For each sensor, 16 features were extracted, and the sample of 96-dimensional eigenvector was formed (6 sensors × 16 features).

In order to comprehensively introduce the data sets that used, the number of attributes, samples and other important information of the three data sets are summarized in Table 4.

## 4. Result and Discussion

The detail of the feature selection algorithms that were used for comparison is described in Section 4.1. Section 4.2 compares the proposed algorithm with the well-known algorithms on multiple assessment indicators over three data sets. In Section 4.3, the effect and superiority of proposed binary transform approaches on GWO based feature selection algorithm will be discussed. Some useful information will be provided in Section 4.4 by analyzing the adaptive restart approach.

### 4.1. Description of the Compared Algorithm

In the experiment, except for the original BGWO, two widely used algorithms for gas sensor data feature selection and two efficient meta-heuristic algorithms were also used to compare with the proposed algorithm, which is summarized as:

Support Vector Machine-Recursive Feature Elimination (SVM-RFE) [42],

Max-Relevance and Min-Redundancy (mRMR) [43],

Binary Grey Wolf Optimization (BGWO) [27],

Discrete Binary Particle Swarm Optimization (BPSO) [44],

Genetic Algorithm (GA) [45].

KNN is a classifier with few parameters and high classification accuracy, so it was used as a wrapper method of all meta-heuristic algorithms in this study; the experimental results show that the classification achieves the best performance when *k* is 5. Each data set was divided into train set and test set with the ratio of 7:3. The error rate of the test set and the number of selected features were used to guide the search direction of all meta-heuristic algorithms, and all of them have been run independently 20 times. mRMR and SVM-RFE are calculated on the complete datasets. Gaussian kernel and linear kernel were used in SVM-RFE, and the optimal feature subset selected from the two kernel functions was taken as the final result. The parameter settings for all algorithms are outlined in Table 5. All parameters were set according to multiple experiments and relevant literature to ensure the fairness of the experiment. The 10-fold cross-validation was conducted on all algorithms in order to eliminate the influence of over fitting.

### 4.2. Comparison of the Proposed Algorithm and Other Algorithms

In the first experiment, adaptive restart GWO with two binary transform approaches (ARGWO1 and ARGWO2) which have been proposed in Section 2 were compared with five feature selection algorithms (mRMR, SVM-RFE, BPSO, GA, BGWO) on three electronic nose datasets.

KNN [46], SVM [47], and Random Forest (RF) [48] were used to calculate the classification accuracy of the feature subset selected by each algorithm to ensure the reliability of accuracy evaluation. Each data set was randomly divided into the training set and testing set with the ratio of 7:3, the classification accuracy of test set was used for the evaluation of feature subset to prove its future performance on the unseen data. The feature subset which gets the least number of features on the premise of the maximum classification accuracy under multiple runs was regarded as the optimal result of each algorithm. The classification accuracy of the optimal feature subset selected by each algorithm on three data sets is as shown in Table 6. We can see that ARGWO1 and ARGWO2 achieve excellent performance: ARGWO2 achieves the highest average classification accuracy on all data sets and the average classification accuracy of ARGWO1 on dataset2 and dataset3 is only less than ARGWO2. In order to judge whether there is over fitting in different models, the accuracy of training sets under different training models is as shown in Table 7, and we can remark that the training was not overfitted.

In order to evaluate the feature subset more comprehensively in classification performance, F1-score was used in this experiment. F1-score is the harmonic mean of precision and recall rate [49], and it can be formulated as in Equation (19):

(19) $$F1=\frac{1}{C}\times \sum_{k=1}^{C}\frac{2\times Pre_{k}\times Rec_{k}}{Pre_{k}+Rec_{k}}$$

(20) $$Pre_{k}=\frac{TP}{TP+FP}$$

(21) $$Rec_{k}=\frac{TP}{TP+FN}$$

where *F*1 is the *F*1-score obtained by each classifier under each data set, *TP* is the number of samples that is correctly predicted, *FP* is the number of samples that errors predicted as class *k*, *FN* is the number of samples belonging to class *k* but is predicted by other classes. The *F*1-score of the optimal feature subsets selected by each algorithm are outlined in Table 8. We can see that ARGWO2 and ARGWO1 achieve the first and second best average *F*1-score. From Table 6 and Table 8, we can see that the classification performance of dataset1 on different classifiers is far lower than other datasets, which is mainly due to the impact of sensor drift on classification accuracy. The experimental results show that selecting appropriate features through the feature selection algorithm can suppress the impact of sensor drift in a certain extent, and the proposed algorithm achieves positive performance in compensating the drift effect.

Table 9 shows the length of the optimal feature subset selected by each algorithm. We can see that the ARGWO2 achieves the minimum average length of the eigenvector. In fact, the classification performance is more important than the length of the feature subset in the assessment indicators system of the feature selection algorithm. Therefore, in the process of determining the optimal result from each algorithm, the classification performance was given priority, and the shortest one was chosen in the feature subsets with the highest classification performance, which also explains the reasons for setting the values of parameters α and β in the fitness function. Table 10 outlines the Wilcoxon test calculated on the classification accuracy and average fitness obtained by the different algorithms. In this experiment, the average classification accuracy of the feature subsets obtained by multiple runs under each dataset and each classifier was regarded as the individual element presented to the Wilcoxon test, and we can remark that the ARGWO2 achieves a significant enhancement over most of the other approaches.

From the above experiments, we can conclude that the proposed algorithm outperforms other methods in classification performance and number of selected features. In addition, ARGWO2 achieves less features while obtaining the highest classification performance on all data sets, which indicates that ARGWO2 can achieve a positive performance on the data collected by different types of electronic nose. By using the proposed algorithm for feature selection, the useful information can be extracted from the gas response signal to enhance the performance of the electronic nose. The feature subset selected by the algorithms with the KNN wrapper method has a similar classification accuracy ranking on three classifiers, which proves that KNN is an effective wrapper method of the meta-heuristics algorithm. The effect of the two proposed mechanisms on the GWO based feature selection algorithm will be discussed in the following subsections.

### 4.3. The Effect of Binary Transform Approach on the Proposed Algorithm

Section 4.2 shows that the proposed algorithm outperforms the original BGWO algorithm in classification performance and the number of selected features. In this section, GWO with sigmoid function (BGWO), GWO with approach1 (GWO1), and GWO with approach2 (GWO2) were used to study the effect of binary transform approach on the GWO for feature selection. In order to control the variables, GWO1 and GWO2 did not add the adaptive restart approach in this experiment. Fitness value is a comprehensive evaluation of the accuracy and length of feature subsets to guide the search direction of the proposed algorithm; thus, it is an important index for the evaluation of the GWO based algorithm, and three fitness related assessment indicators were used in this experiment [27]:

The best fitness is the minimum fitness value obtained by running the algorithm for *M* times, and it can be formulated in Equation (22):

(22) $$f_{best}=\min_{i}^{M}f_{i}$$

The worst fitness is the maximum fitness value obtained by running the algorithm for *M* times, and it can be formulated in Equation (23):

(23) $$f_{worst}=\max_{i}^{M}f_{i}$$

The mean fitness is the average fitness value obtained by running the algorithm for *M* times, and it can be formulated in Equation (24).

(24) $$f_{mean}=\frac{1}{20}\sum_{i=1}^{M}f_{i}$$

**Figure 2:**
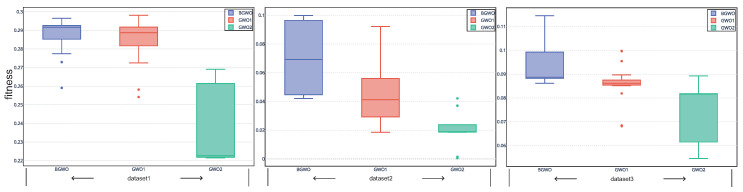
Boxplots for the fitness value obtained from different algorithms.

**Figure 3:**
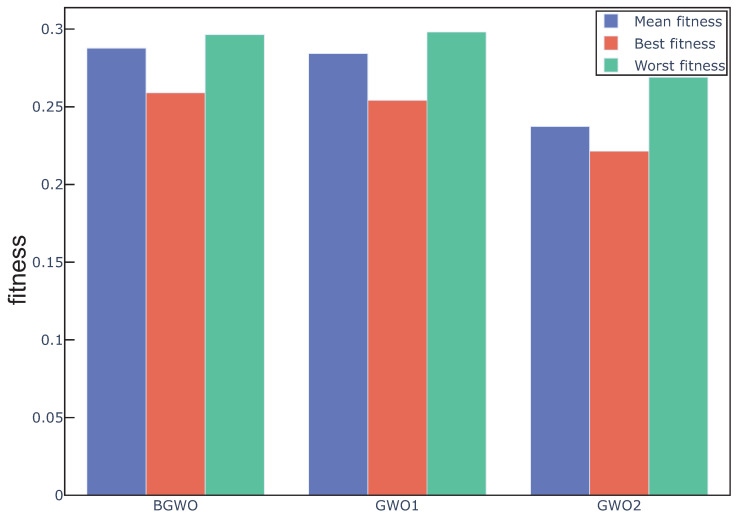
Mean, best, and worst fitness obtained from different algorithms over dataset1.

**Figure 4:**
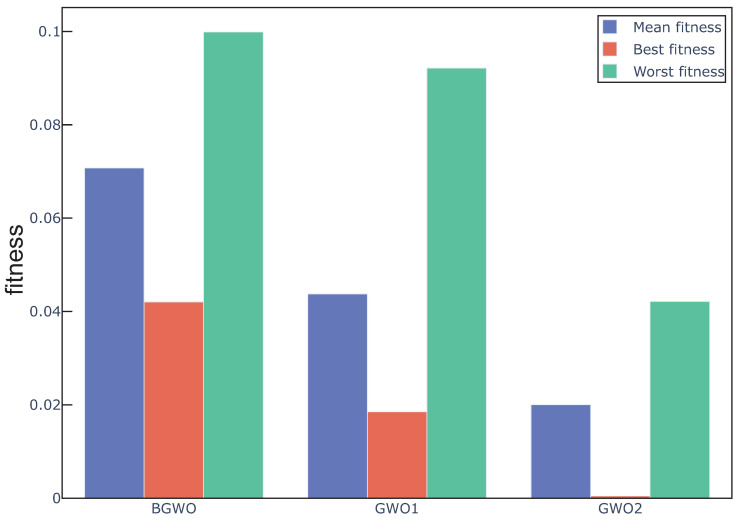
Mean, best, and worst fitness obtained from different algorithms over dataset2.

**Figure 5:**
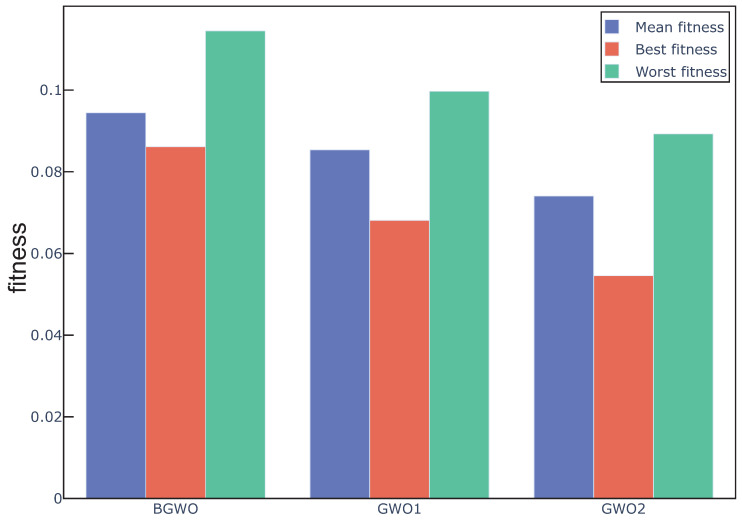
Mean, best, and worst fitness obtained from different algorithms over dataset3.

Figure 2 shows the fitness value obtained by three algorithms in 20 independent runs over all the datasets. In addition, according to Equation (10), we can remark that the feature subset with lower fitness, which means lower error rate and fewer selected features, represents the better search performance. We can see from the figure that the overall performance of GWO1 and GWO2 is better than BGWO. Figure 3, Figure 4 and Figure 5 outline the best, mean, and worst fitness value obtained by three algorithms over all the data sets. We can see that GWO1 has a slight advantage over BGWO. In addition, GWO2 achieves much better performance than the other two algorithms. The Wilcoxon test was used to verify the significant difference between the above algorithms, and the average fitness over three datasets was regarded as the individual element presented to the Wilcoxon test, and the average fitness values under multiple runs were used to calculate the *p*-value between different algorithms. From the experiment results, we can remark that the GWO1 and GWO2 achieve significant enhance over the BGWO by achieving the *p*-value of 0.0081 and 0.0076. We can remark that the binary transform approach has a positive influence on the search capability of GWO for feature selection, and it is more advantageous to find the optimal feature subset by using the appropriate approaches. Compared with sigmoid function, the search capability of the algorithm can be improved more by approach1 or approach2. Moreover, approach2 achieves an outstanding advantage, and it is possible that approach2 takes into account the information on the position vector of search agents rather than relying on only one element of the position vector in the process of binary transformation. Therefore, we take the attitude that it may be a good way to control the binary transform process by combining more information related to search agents.

### 4.4. The Effect of Adaptive Restart Approach on the Proposed Algorithm

**Figure 6:**
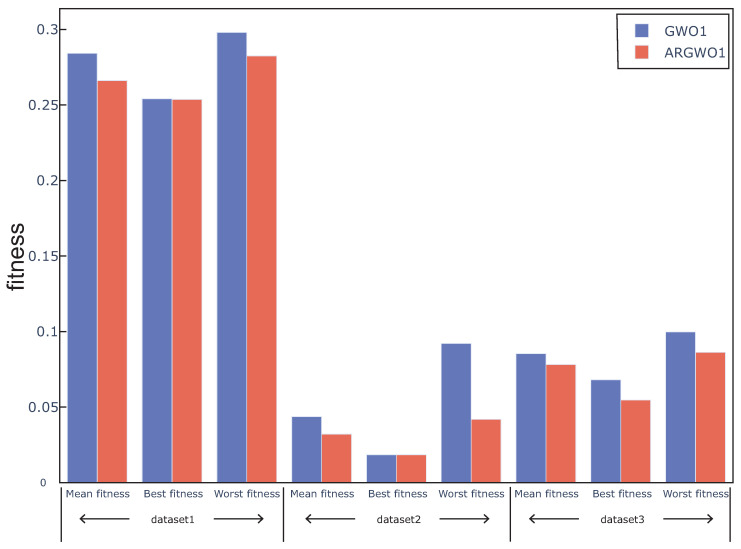
Mean, best, and worst fitness obtained from GWO1 and ARGWO1 over all datasets.

**Figure 7:**
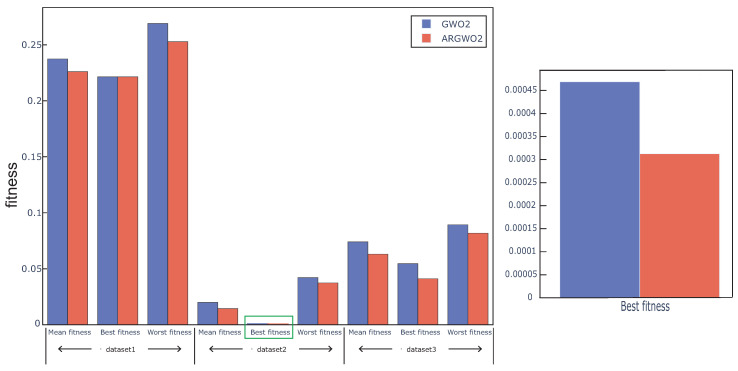
Mean, best, and worst fitness obtained from GWO2 and ARGWO2 over all datasets. The inset shows an expanded plot of the area in the green rectangle.

In this section, GWO with an adaptive restart approach(ARGWO1, ARGWO2) and without an adaptive restart approach (GWO1, GWO2) were used to study the effect of the adaptive restart approach on the algorithm. Figure 6 and Figure 7 show the best, worst, and average fitness value obtained by the above four algorithms over all the data sets. We can see that the mean fitness and the worst fitness of the algorithm are reduced by using an adaptive restart approach, and the best fitness is also reduced in some cases, which proves that the adaptive restart approach effectively improves the search capability. The Wilcoxon test was also used in this experiment, the average fitness over three datasets was regarded as the individual element presented to the Wilcoxon test, and the average fitness values under multiple runs were used to calculate the *p*-value between the proposed algorithm with and without adaptive restart. The *p*-value between ARGWO1 and GWO1 achieved 0.0288, and the *p*-value between ARGWO2 and GWO2 achieved 0.0036. We can remark that the performance of the proposed algorithm is significantly improved by using adaptive restart.

**Figure 8:**
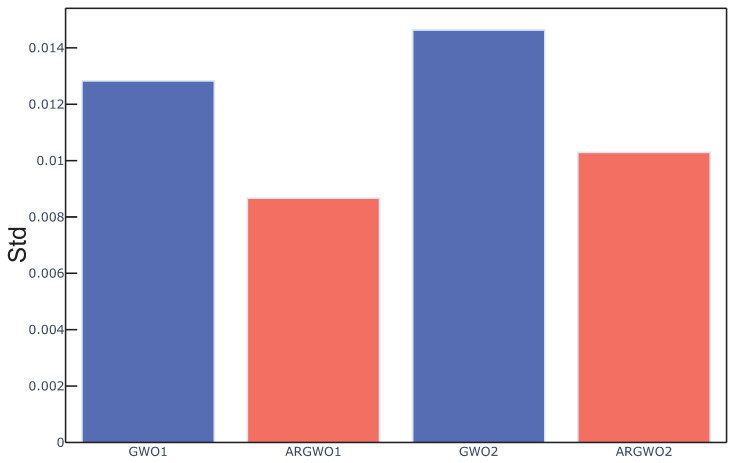
Std measure average over all the data sets for the different algorithms.

Std is a measure for the variation of the optimal result obtained by the algorithm under multiple runs [50]. In addition, it was used as the index to evaluate the stability of the algorithm in this experiment. *Std* is formulated as in Equation (25).

(25) $$Std=\sqrt{\frac{1}{20}\sum_{i=1}^{20}{\left(f_{i}-f_{mean}\right)}^{2}}$$

where $f_{i}$ is the final fitness value of the independent operation *i*, $f_{mean}$ is the mean fitness. The average *Std* value over all data sets obtained by the above four algorithms in this experiment are outlined in Figure 8. We can see from the figure that the stability and repeatability of the GWO based algorithm can be improved by using an adaptive restart approach. The adaptive restart approach is based on the optimal fitness value and the number of search agents in each iteration to determine the number of search agents that need to be reinitialized. By analyzing the current search effect, adaptive restart can dynamically affect the search direction of the algorithm when the algorithm has already or has a tendency to fall into the local optimum, so as to prevent falling into the local optimal solution and select more useful features from the gas response signal.

From all these experiments, we can conclude that the proposed algorithm outperforms other algorithms over all datasets, which indicates that the proposed algorithm can be applied to different types of electronic nose and effectively enhance their performance of gas sensing. The performance of the GWO based algorithm for feature selection of electronic nose data are effectively improved by proposed binary transform approaches and adaptive restart. The fitness value of the selected feature subset can be reduced by using proposed binary transform approaches, especially in approach2. The stability and search capability of the GWO based feature selection algorithm can be further enhanced by using adaptive restart approach. Throughout the paper, the proposed algorithm can effectively select more favorable feature subsets for gas recognition, but, because the adaptive restart approach does not add too much influence to the search behavior of GWO, there is still a certain probability of falling into local optimum in the process of searching. In order to obtain the optimal feature subsets, it is usually necessary to run multiple times.

## 5. Conclusions

This paper proposes a novel method for enhancing the performance of electronic nose by feature selection using the improved grey wolf optimization based algorithm. Two novel binary transform approaches and adaptive restart were employed for the proposed algorithm. The proposed algorithm was compared with five typical feature selection algorithms on three electronic nose datasets. Three classifiers and multiple assessment indicators were used to evaluate the performance of each algorithm. The results showed that the proposed algorithm outperformed original BGWO and other algorithms. In addition, the search capability of the GWO can be effectively improved by using adaptive restart and proposed binary transform approaches. The proposed binary transform approaches can search feature subset from electronic nose data that maximizes the classification accuracy while minimizing the number of features and adaptive restart attempts to further enhance the search capability and the stability of the algorithm, which will help us to obtain more favorable feature subsets for pattern recognition from high-dimensional electronic nose data. In summary, the proposed algorithm is a promising method for enhancing classification accuracy and reducing dimension, which can effectively enhance the performance of different types of electronic nose. In the future, the proposed algorithm can be combined with more kinds of features to improve the performance. 

## Figures and Tables

**Table 1:** The details of dataset1.

No.	Gas	No. Samples	Concentration Range (ppmv)
1	Ammonia	1641	(50, 1000)
2	Acetaldehyde	1936	(5, 500)
3	Acetone	3009	(12, 1000)
4	Ethylene	2926	(10, 300)
5	Ethanol	2565	(10, 600)
6	Toluene	1833	(10, 100)

The value of each number in the column called “No. samples” is the number of samples obtained in each gas environment. Dataset1 collects the data of six kinds of gases, and the total number of samples obtained in six kinds of gases is 13,910.

**Table 2:** Feature description for ordinary sensors in dataset2 and dataset3.

Feature Type	Descripition
Maximum response	Max response value of curve-baseline
Derivative	Maximum and minimum derivative of sensor value
Time constant	The time when the sensor value reaches the 30%, 60%, and 90% of its maximum response
Integral	The integral of curve calculated by $I=\int_{T_{gasOn}}^{T_{gasOff}}\;\left(x\left(t\right)-baseline\right)\;dt$
Equal interval value	Obtain ten values in the sensor data at equal time intervals from $T_{gasOn}$ to $T_{gasOff}$

**Table 3:** Gas sensors array setup of dataset3.

Number	Sensors	Description	Load Resistance (kΩ)
1,4	MQ-3	Small sensitivity to Benzine and high sensitivity to alcohol	22
2,5	MQ-4	High sensitivity to natural gas and CH4	18
3,6	MQ-6	High sensitivity to iso-butane, LPG, propane	22

**Table 4:** Summary of all data sets.

Number	Description	No. Attributes	No. Samples	No. Classes
1	Detection of gases of different concentrations	128	13,910	6
2	Gas sensor array exposed to turbulent gas mixtures	128	180	2
3	Wine quality inspection	96	300	4

**Table 5:** Parameter setting.

Parameter	Value
Number of search agents	40
Number of max iterations	30
Dimension	Number of features
*k* neighbors of KNN	5
α of the fitness function	0.99
β of the fitness function	0.01
Variation fraction of GA	0.02
$c_{1}$, $c_{2}$ of BPSO	2
C parameter of SVM-RFE	2
γ parameter of SVM-RFE	0.2

**Table 6:** Classification accuracy comparison of various methods in the test set.

Algorithm	Dataset1				Dataset2				Dataset3			
	KNN	SVM	RF	Average	KNN	SVM	RF	Average	KNN	SVM	RF	Average
mRMR	0.6393	0.7128	0.6531	0.6684	0.8889	**0.9815**	0.9074	0.9259	0.8493	0.8904	0.8630	0.8676
SVM-RFE	0.7559	**0.8317**	0.6485	0.7454	0.8889	0.9444	0.8704	0.9012	0.8630	0.9041	0.8630	0.8767
GA	0.7332	0.7438	0.6746	0.7172	0.9630	0.9630	0.9074	0.9444	0.9178	0.9041	0.8904	0.9041
BPSO	0.7550	0.7275	0.6043	0.6956	0.9630	0.9630	0.8889	0.9383	0.9041	0.9041	0.8767	0.8950
BGWO	0.7430	0.7504	0.6045	0.6993	0.9630	0.9630	0.8889	0.9383	0.9178	0.9041	0.8904	0.9041
ARGWO1	0.7487	0.8104	0.6539	0.7377	0.9814	0.9259	**0.9444**	0.9506	0.9315	0.9041	**0.9041**	0.9132
ARGWO2	**0.7769**	0.8145	**0.6801**	**0.7572**	**1.0000**	**0.9815**	**0.9444**	**0.9753**	**0.9452**	**0.9178**	**0.9041**	**0.9224**
FULL	0.6545	0.6488	0.6252	0.6428	0.8519	0.9259	0.8889	0.8889	0.7671	0.8767	0.8767	0.8402

**Bold** values indicate the best results.

**Table 7:** Classification accuracy comparison of various methods in the training set.

Algorithm	Dataset1			Dataset2			Dataset3		
	KNN	SVM	RF	KNN	SVM	RF	KNN	SVM	RF
mRMR	0.7663	0.8646	0.7676	0.9206	0.9841	0.9524	0.9333	0.9523	0.9667
SVM-RFE	0.8121	0.8629	0.7172	0.9285	0.9841	0.9047	0.9554	0.9813	0.9381
GA	0.8084	0.8770	0.7498	0.9365	0.9206	0.9047	0.9554	0.9420	0.9667
BPSO	0.8138	0.8660	0.7044	0.9444	0.9683	0.9047	0.9420	0.9381	0.9523
BGWO	0.7965	0.8908	0.7211	0.9683	0.9841	0.8902	0.9554	0.9420	0.9667
ARGWO1	0.8162	0.8953	0.7049	0.9841	1.0000	0.9683	0.9420	0.9420	0.9420
ARGWO2	0.8215	0.8768	0.7398	0.9841	0.9841	0.9683	0.9824	0.9912	0.9420
FULL	0.7847	0.7049	0.7153	0.8730	0.9365	0.8902	0.8512	0.8845	0.9420

**Table 8:** F1-score comparison of various methods.

Algorithm	Dataset1	Dataset2	Dataset3	Average
mRMR	0.6750	0.9254	0.7866	0.7957
SVM-RFE	0.7376	0.9009	0.7697	0.8027
GA	0.7193	0.9442	0.8571	0.8402
BPSO	0.6911	0.9380	0.8438	0.8243
BGWO	0.6951	0.9380	0.8583	0.8305
ARGWO1	0.7324	0.9503	0.8572	0.8466
ARGWO2	**0.7597**	**0.9751**	**0.8770**	**0.8624**
FULL	0.6466	0.8880	0.7746	0.7697

**Bold** values indicate the best results.

**Table 9:** Features number comparison of various methods.

Algorithm	Dataset1	Dataset2	Dataset3	Average
mRMR	25	25	36	28.6667
SVM-RFE	27	32	19	26
GA	51	67	55	57.6667
BPSO	75	75	48	66
BGWO	59	70	55	61.3333
ARGWO1	69	39	**7**	38.3333
ARGWO2	**8**	**20**	10	**12.6667**
All	128	128	96	117.3333

**Bold** values indicate the best results.

**Table 10:** *p*-value between the ARGWO2 and other approaches.

Algorithm	Factor	mRMR	SVM-RFE	GA	BPSO	BGWO	ARGWO1
ARGWO2	Accuracy	0.0199	0.0358	0.0268	0.0082	0.0085	0.1326
	Average fitness	0.0072	0.0127	0.0193	0.0024	0.0039	0.1103

## Abbreviations

The following abbreviations are used in this manuscript:

GWO	Grey Wolf Optimizer
KNN	k-Nearest Neighbor
SVM-RFE	Support Vector Machine-Recursive Feature Elimination
mRMR	Max-Relevance and Min-Redundancy
BGWO	Binary Grey Wolf Optimization
GA	Genetic Algorithm
SVM	Support Vector Machine
